# A one-step hydrothermal route to fabricate a ZnO nanorod/3D graphene aerogel-sensitized structure with enhanced photoelectrochemistry performance and self-powered photoelectrochemical biosensing of parathion-methyl†

**DOI:** 10.1039/d1ra06339a

**Published:** 2021-11-03

**Authors:** Yuting Yan, Qian Li, Qirui Wang, Hanping Mao

**Affiliations:** School of Agricultural Engineering, Jiangsu University Zhenjiang 212013 People's Republic of China yanyuting@ujs.edu.cn maohp@ujs.edu.cn +86 511 88797338 +86 511 88797338; School of Food Science and Engineering, Yangzhou University Yangzhou 225127 People's Republic of China; School of Aeronautical and Mechanical Engineering, Changzhou Institute of Technology Changzhou 213022 People's Republic of China

## Abstract

Developing a new functional sensitized structure for improving the inherent property of photoactive materials and selecting an efficient electron donor as a photoelectrochemical (PEC) signal amplification strategy are crucial for achieving excellent PEC biosensors. Herein, we present a facile one-pot hydrothermal strategy for fabricating ZnO nanorod-decorated 3D graphene aerogels (ZnO/GAs). In this nanoarchitecture, GAs act as a new generation enhanced carrier, which can effectively improve the photoactivity of ZnO under visible light by enhancing the interfacial charge transfer. In addition, the as-prepared ZnO/GA nanocomposites exhibited two times higher photocurrent intensity than that of ZnO/graphene. Furthermore, we developed a novel self-powered PEC biosensor based on a ZnO/GAs sensitized structure with the amplification of enzymolysis products for the detection of parathion-methyl. Thiocholin, as a sacrificial electron donor, which is produced from the hydrolysis of acetylthiocholine catalyzed by acetylcholinesterase (AChE), can further significantly improve the photocurrent. Then, the inhibition of AChE activity by parathion-methyl prevented the production of thiocholine, resulting in an obvious decrease in photocurrents. Based on the above results, we fabricated an AChE-based self-powered PEC biosensor for the sensitive and selective determination of parathion-methyl with a linear range of 0.1 ng mL^−1^ to 0.1 μg mL^−1^ and a detection limit of 0.03 ng mL^−1^ (S/N = 3). This PEC biosensing strategy not only gives insights into using GAs as a novel sensibilizer to improve the PEC nature of semiconductors but also provides a promising flexible platform for organophosphorus pesticide detection.

## Abstract 2

A facile one-pot hydrothermal method for fabricating ZnO/GAs was developed. And a novel self-powered PEC biosensor was constructed based on the ZnO/GAs with the amplification of thiocholine for the detection of parathion-methyl.

## Graphical abstract

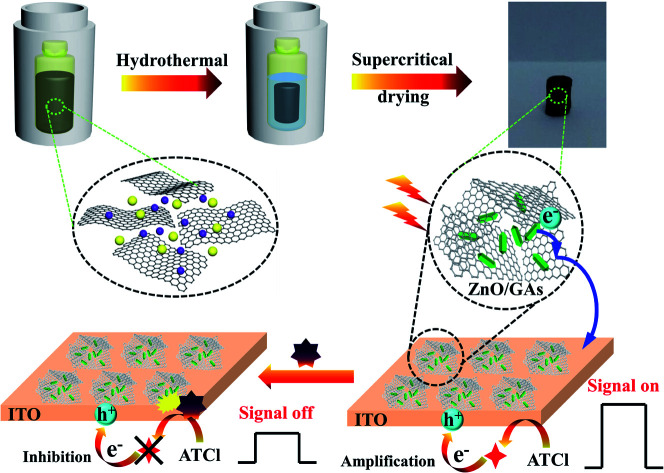

## Introduction

Photoelectrochemical (PEC) biosensors, as emerging devices, have aroused extensive attention in biological analysis,^1,2^ environment monitoring^3^ and food safety,^4^ due to their advantages of easy operation, high accuracy and low cost.^5–7^ Among them, the self-powered PEC biosensors, without providing any extra voltage, take the advantages of making sufficient use of visible light and void the interference of oxidation/reductive substances, and have attracted wide attention. In this system, the electron transfer efficiency of photoactive species and the biological reaction are crucial to the PEC analytical performance. Thus, developing new functional sensitized structure with high PEC conversion efficiency as photoactive materials is a major way to improve the performance of self-powered PEC biosensors. On the one hand, some new PEC transducers have been designed, for example, Yang *et al.*^8^ reported the noncovalent coupling of C60 to an electronically complementary porphyrin-derived metal–organic framework (PCN-224) with carboxyl-group terminals as photocurrent enhanced materials, and then, this new photocurrent enhanced material was applied to a PEC immunosensor for S100 calcium-binding protein B. On the other hand, a great deal of research is focused on fabricating traditional PEC semiconductor material-based functional sensitized nanostructures, such as ZnO,^9,10^ TiO_2_,^11^ CuFe_2_O_4_,^12^ BiOI^13^ and carbon nitride,^14,15^ for self-powered PEC biosensors. ZnO has drawn great attention due to its good stability, low cost and environment friendliness. Nevertheless, the wide bandgap (∼3.3 eV) makes it absorb only in the ultraviolet range, and the fast recombination of the electron–hole pairs also limits its application in PEC transducers.^16,17^ To develop ZnO PEC transducers, various sensitizers, including atomic,^18^ dye,^19^ heterojunction^20–22^ and graphene-based materials,^23,24^ have been doped for improving the PEC intensity of ZnO and extending its photo response to the visible region. Graphene-based material-sensitized structures dynamically develop an efficient path to improve the PEC performance of ZnO due to their outstanding electrical conductivity.

Graphene aerogels (GAs), as three-dimensional graphene, can overcome the aggregation and stack of graphene sheets caused by the strong π–π interactions.^25–29^ Thus, GAs exhibit strong mechanical strength and high transfer rate for mass and electrons due to the admirable intrinsic properties and the stable interconnected framework of graphene.^29–31^ Most recently, GAs are expected as a new generation enhanced carrier in the field of electrochemistry and photocatalysis due to the enhancing conductivity, high specific area and fast separation of photo-generated electrons and holes. For example, Fan *et al.*^32^ fabricated sub-micrometer composites (ZnO@GAs) as anodes in lithium-ion batteries by the one-pot solvothermal strategy, and they found that the GAs framework can enhance the electrical conductivity and buffer any volume expansion, contributing to a high capacity and cyclic stability of ZnO@GAs. The result also reflects the great potential of GAs as a ESI† for electrochemistry. Wei *et al.*^33^ demonstrated that three-dimensional graphene/ZnO nanorods show better performance in photocatalytic degradation towards methyl orange under UV light than pure ZnO nanorods due to the effective separation of photo-generated electrons and holes at the interface of three-dimensional graphene and ZnO. The result reflects the possible potential of GAs as enhanced carriers for PEC transducers. According to Yue's report,^34^ ZnO nanowire arrays were vertically grown on a three-dimensional graphene foam used as an electrochemical electrode for the sensitive and selective determination of levodopa. The excellent electrical conductivity and large surface areas of graphene foam endow the biosensor with outstanding performance, implying the potential application of GAs in biosensors. However, these above-mentioned three-dimensional graphene-based sensitized structures were generally synthesized by two steps: the three-dimensional graphene was synthesized by chemical vapor deposition first, and then, incorporated with ZnO by a hydrothermal method, which was a complicated and time-consuming procedure. Thus, it is extremely necessary to design such a facile synthesis method to obtain three-dimensional graphene-based sensitized structures and to open up new prospects for the utilization of three-dimensional graphene as a novel enhanced material in various applications.

Of course, except for designing appropriate sensitized structures as photoactive materials, selecting an efficient electron donor produced by a biological reaction is also crucial to the PEC signal amplification. Thiocholine, produced from the hydrolysis of acetylthiocholine catalyzed by acetylcholinesterase (AChE), has been demonstrated as an ideal candidate for signal amplification in PEC biosensors.^35^ Moreover, the selectivity and specificity of the enzyme also guarantee the inherent sensitivity of the PEC enzyme biosensor.

Here, we describe a facile one-pot hydrothermal method for fabricating ZnO nanorod-decorated 3D graphene aerogels (ZnO/GAs). GAs were employed as the sensitizer, which could efficiently improve the PEC performance of ZnO than that of graphene. Furthermore, a novel self-powered PEC biosensor was constructed based on the ZnO/GAs sensitized structure with the amplification of enzymolysis products (thiocholine) for the detection of parathion-methyl. Based on the inhibition of AChE activity by parathion-methyl, a proposed AChE-based biosensor could be applied to the monitoring of the parathion-methyl detection. This PEC biosensor showed a satisfactory performance with a rapid response and low detection, providing a flexible platform for pesticide detection.

## Experimental

### Reagents

Chemically pure Zn(NO_3_)_2_·6H_2_O, NaH_2_PO_4_ and Na_2_HPO_4_ were purchased from Sinopharm Chemical Reagent Co., Ltd. Acetylcholinesterase (AChE, Type C3389, 500 U mg^−1^ from electric eel) and acetylthiocholine chloride (ATCl) were purchased from Sigma-Aldrich (USA). Parathion-methyl, acetamiprid and pentachlorophenol were purchased from Aladdin Chemistry Co., Ltd. Graphite was purchased from Qingdao Tianhe Graphite Co., Ltd. 0.1 M PBS with the pH value of 7.4 was employed as the electrolyte buffer, which was prepared with standard solutions of NaH_2_PO_4_ and Na_2_HPO_4_, and adjusted the pH with 0.1 M H_3_PO_4_ or NaOH solution. Other reagents were of analytical grade and used as received without further purification, and all solutions were prepared with ultrapure water (18 MΩ) from a Milli-Q water purification system.

### Apparatus

Transmission electron microscopy (TEM) image was taken with a JEOL 2100 transmission electron microscopy (JEOL, Japan) operated at 200 kV. X-ray diffraction (XRD) analysis was conducted on a Bruker D8 diffractometer with high-intensity Cu Kα (*λ* = 1.54 Å). X-ray photoemission spectroscopy (XPS) was recorded on a VG MultiLab 2000 system with a monochromatic Mg-Kα source operated at 20 kV. All the electrochemical and PEC measurements were conducted using a CHI660 B electrochemical analyzer (Chen Hua Instruments, Shanghai, China). A traditional three-electrode system was established with a ITO conducting glass (1 cm × 0.5 cm), a Pt wire and Ag/AgCl (saturated KCl solution) as the working, counter and reference electrodes, respectively. A 250 W Xe lamp (Beijing Trusttech Co. Ltd) was used as the visible light source with an intensity (passing through a 400 nm UV-cut filter) of 100 mW cm^−2^. Electrochemical impedance spectroscopy (EIS) was performed in a 0.1 M KCl solution containing 5 mM Fe(CN)_6_^3−/4−^ with a frequency range from 0.01 Hz to 10 kHz, and the amplitude of the applied sine wave potential in each case was 5 mV.

### Preparation of ZnO/GAs, ZnO/graphene (ZnO/GR) nanocomposites and ZnO

**Scheme 1:**
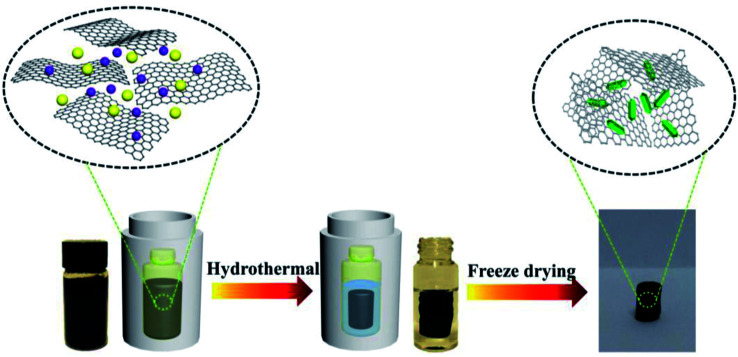
Schematic of the one-pot hydrothermal strategy for fabricating ZnO/GAs nanocomposites.

First, graphene oxide (GO) was prepared from natural graphite according to a modified Hummer's method.^36,37^ZnO/GA nanocomposites were prepared as follows (Scheme 1): 50 mg of GO was uniformly dispersed in 10 mL of ultrapure water *via* ultrasonication, and then, 5 mg of Zn(NO_3_)_2_·6H_2_O was added. The mixed solution was sonicated for 30 min to obtain a homogeneous aqueous dispersion. The above-mentioned homogeneous-mixed aqueous dispersion was put in a glass bottle, sealed in a 20 mL Teflon-lined autoclave, and maintained at 160 °C for 12 h. After cooling to room temperature, the as-prepared ZnO/GAs were taken out by a tweezer, and washed with ultrapure water and a 10% aqueous solution of ethanol for several times. After freeze-drying at −80 °C for 12 h, black-integrated ZnO/GAs were obtained. GAs were obtained under the same condition without Zn(NO_3_)_2_·6H_2_O. ZnO/GR nanocomposites were obtained as follows: 50 mg of GO was uniformly dispersed in 10 mL of ultrapure water *via* ultrasonication, and then, 5 mg of Zn(NO_3_)_2_·6H_2_O was added. The mixed solution added with 0.1 M NaOH, and sonicated for 30 min to obtain a homogeneous aqueous dispersion. The above-mentioned homogeneous-mixed aqueous dispersion was sealed in a 20 mL Teflon-lined autoclave without putting in a glass bottle, and maintained at 160 °C for 12 h. After cooling to room temperature. After cooling to room temperature, the as-prepared ZnO/GR were washed with ultrapure water and a 10% aqueous solution of ethanol for several times, and dried in vacuum at 45 °C for 24 h to obtain the ZnO/GR. ZnO was obtained under the same condition used that for the ZnO/GR nanocomposites without GO.

### Fabrication of the PEC biosensor

2 mg of the as-prepared ZnO/GAs was dispersed in 10 mL ultrapure water. Prior to the modification of ITO, the ITO electrodes were placed in boiling water containing 0.01 M NaOH for 0.5 h, followed by ultrasonically cleaning in water and alcohol for 30 min, respectively. Afterwards, 20 μL of the ZnO/GAs suspension was dropped onto the surface of the ITO electrode with a fixed area of 0.5 cm^2^ and dried under an infrared lamp to obtain the modified ITO (ZnO/GAs/ITO). For comparison, ZnO, GAs and ZnO/GR modified ITO were also prepared in the same way (ZnO/ITO, GAs/ITO, and ZnO/GR/ITO). The obtained ZnO/GAs/ITO electrode was finally coated with a 6 μL AChE solution (293 U mL^−1^) and incubated at 25 °C for 30 min. After evaporation of water, the modified ITO was washed with 0.1 M PBS to remove the unbound AChE, and the resulted AChE/ZnO/GAs/ITO was stored at 4 °C when not in use.

## Results and discussion

### Characterization of ZnO/GAs

**Fig. 1:**
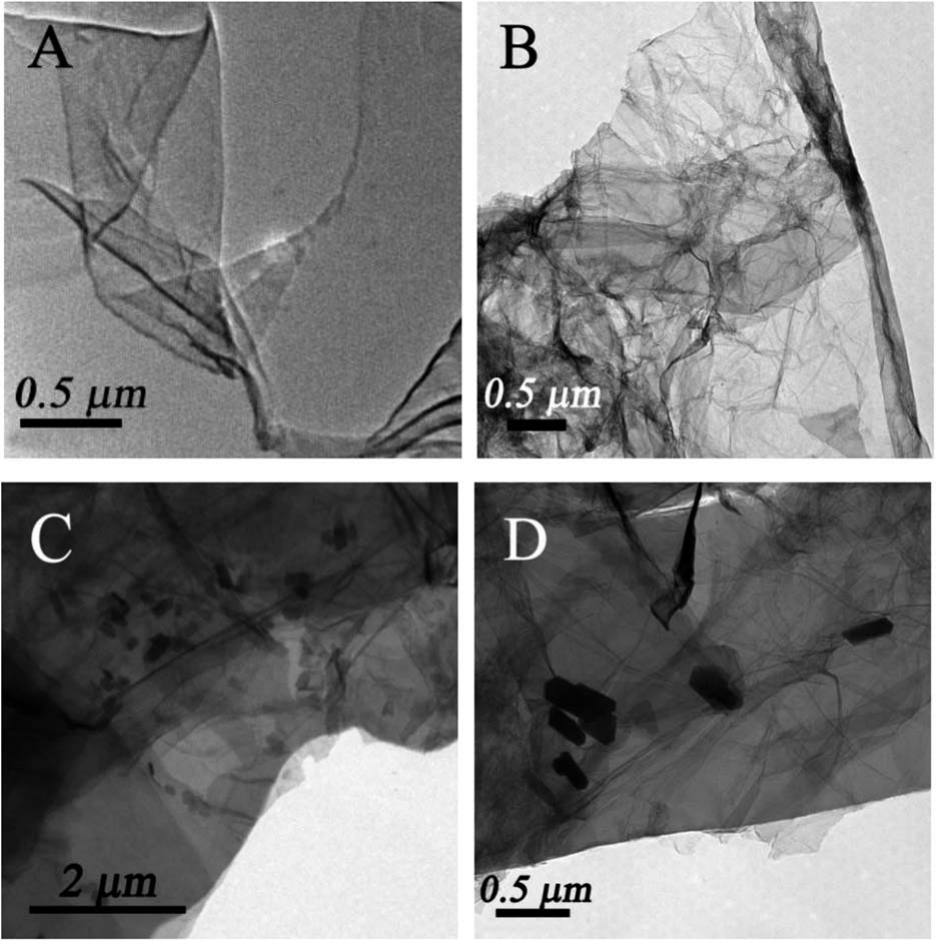
TEM image of GO (A), GAs (B) and ZnO/GAs nanocomposites (C) and (D).

TEM was employed to characterize the morphology of the as-prepared nanocomposites. Fig. 1A displays a typical TEM image of single sheets of graphene. From Fig. 1B, it is clearly observed that numerous wrinkly structures were observed on the surface of GAs because of different levels of transparency, indicating that the three-dimensional structures formed successfully. The SEM image of GAs (Fig. S1†) further reveals that the three-dimensional GAs have a rugged surface with micropores. The TEM image of ZnO/GAs (Fig. 1C) clearly exhibits that the ZnO nanorods were homogeneously distributed on the GAs surfaces, suggesting the assembly between the ZnO nanorods and interconnected 3D network graphene during the hydrothermal treatment. From the high-magnification TEM image (Fig. 1D), ZnO nanorods were in a hexagonal structure, and the diameter of the ZnO nanorods was approximately 60–90 nm with the average length of about 0.2 μm. The EDS spectra of ZnO/GAs nanocomposites is shown in Fig. S2A,† which further confirmed the presence of Zn, C and O, and indicated the successful formation of ZnO/GAs. Besides, the elemental mapping results (Fig. S2B–D†) confirmed that ZnO/GAs had the desired compositions in the form of zinc (Zn), carbon (C) and oxygen (O), which is in accordance with the EDS spectra of the ZnO/GAs nanocomposites.

**Fig. 2:**
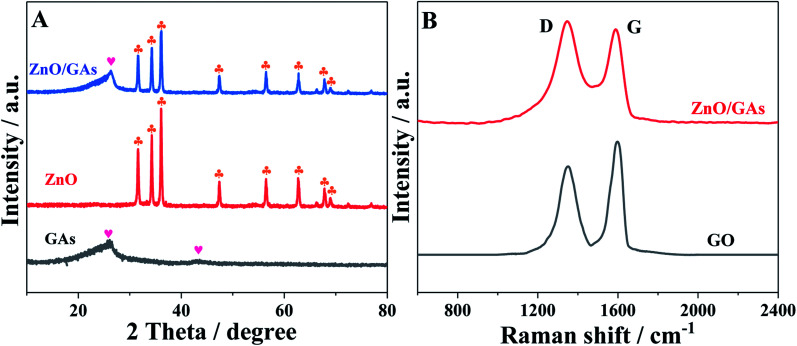
(A) XRD pattern of GAs, ZnO and ZnO/GAs nanocomposites, (B) Raman spectra of GO and ZnO/GAs nanocomposites.

To obtain the phase and crystal structural information of the as-prepared samples, the XRD patterns of GAs, ZnO and ZnO/GAs were obtained, as shown in Fig. 2A. The main diffraction peaks at 31.9°, 34.5°, 36.1°, 47.5°, 56.4°, 62.8° and 68.2° ascribed to (100), (002), (101), (102), (110), (103) and (112) planes of ZnO can be indexed to the wurtzite phase of ZnO (JCPDS, card no: 36-1451),^17,38^ confirming the formation of single-crystalline ZnO during the hydrothermal process. In addition, from Fig. 2A, the two diffraction peaks for the GAs at 2*θ* = 26.3° and 2*θ* = 43.3° are observed, corresponding to the (002) and (100) diffraction of the graphene structure.^34,39^ Compared to the XRD of GO (Fig. S3†), the diffraction peak of graphite (002) increased from 11.2° to 26.3°, indicating that oxygen functional groups have been removed from the GO surface.

From Raman spectroscopy (Fig. 2B), both GO and ZnO/GAs show the existence of D and G peaks located at around 1350 cm^−1^ (D-band) and 1595 cm^−1^ (G-band). The intensity ratio *I*_D_/*I*_G_ of 0.85 in GO has changed to 1.06 in ZnO/GAs, demonstrating that a large number of structural defects have been introduced. Some defects in graphene-based materials are conducive to improve the electrochemical performance.

**Fig. 3:**
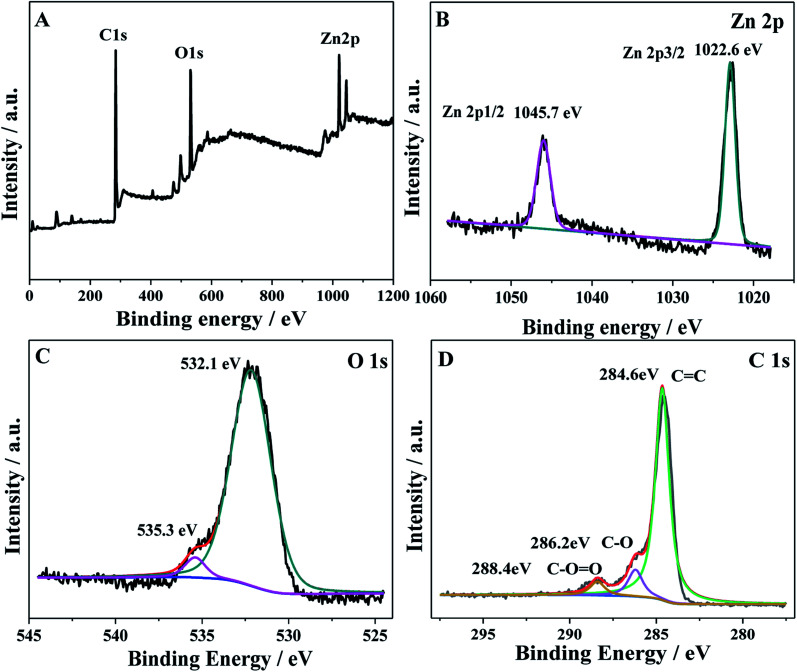
(A) XPS survey spectra of ZnO/GAs nanocomposites; the high-resolution XPS spectra of the (B) Zn 2p, (C) O 1s and (D) C 1s region for ZnO/GAs nanocomposites.

To further investigate the oxidation state and the chemical composition of the as-prepared ZnO/GAs, XPS measurements were performed. As shown in Fig. 3A, the total survey spectrum was centered at the region of 0–1100 eV, corresponding to the characteristic peaks of Zn 2p, O 1s, and C 1s, suggesting the existence of Zn, O and C elements, respectively. In the high-resolution XPS spectrum of Zn 2p (Fig. 3B), two strong peaks with binding energies at 1045.7 and 1022.6 eV were consistent with the XPS data of Zn 2p_1/2_ and Zn 2p_3/2_, respectively.^40^ The XPS spectra of O 1s for ZnO/GAs is shown in Fig. 3C, and the peak at 532.1 eV is associated with the original lattice oxygen species of ZnO.^41^ Besides, the peak at 535.3 eV was associated with –OH.^42^Fig. 3D reveals the high resolution C 1s spectra of ZnO/GAs, which can be resolved into three different peaks at 284.6, 286.2 and 288.4 eV, corresponding to C–C/C=C in aromatic rings, C–O and C=O groups, respectively.^43^ These above-mentioned results are consistent with the XRD, indicating the existence of both ZnO and GAs in the as-prepared nanocomposites.

### PEC behaviors of the ZnO/GAs/ITO

**Fig. 4:**
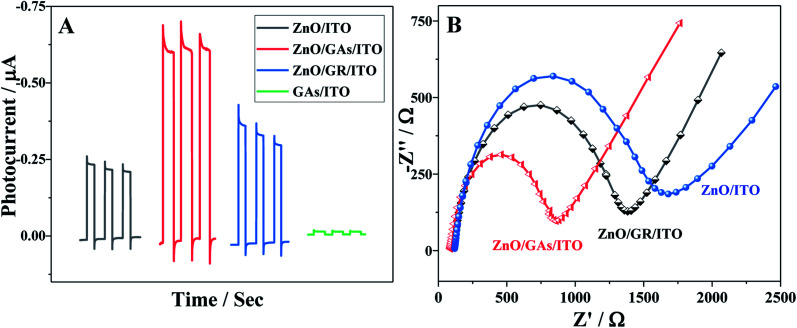
(A) Photocurrent responses of ZnO/ITO, ZnO/GR/ITO, ZnO/GAs/ITO and GAs/ITO; (B) EIS responses of ZnO/ITO, ZnO/GR/ITO, ZnO/GAs/ITO.

The PEC performances of different modified electrodes were evaluated in 0.1 M PBS (pH 7.4) under visible irradiation. As clearly shown in Fig. 4A, no obvious photocurrent is observed for GAs/ITO (curve d), and the ZnO/ITO electrode exhibits an obvious photocurrent response to light illumination, indicating the excellent photocatalytic activity of ZnO. The ZnO/ITO electrode alone exhibited the signal of 0.20 μA, while the PEC response of the ZnO/GAs/ITO electrode appeared to be 3-times higher than that of ZnO/ITO because the introduction of GAs can enhance the light absorption and reduce the charge recombination of the photogenerated electrons and holes. Moreover, to further evaluate the enhancement effect of GAs on the PCE signal of ZnO, the PEC responses of the ZnO/GR electrode were investigated by contrast. It was found that the photocurrent of the ZnO/GAs/ITO electrode was higher than that of the ZnO/GR/ITO electrode, suggesting that GAs showed more efficient sensitization effect than that of GR on the PEC response of ZnO because the GAs interconnected framework exhibits a higher electron transfer rate than that of graphene.

In order to demonstrate this, the interfacial behavior of different modified electrodes were probed by recording the electrochemical impedance spectra (EIS) of [Fe(CN)_6_]^3−/4−^ (Fig. 4B). The semicircle diameter of the Nyquist plot representing the interfacial charge-transfer resistance (*R*_ct_) of the ITO electrode increased after modification with ZnO due to the low conductivity of ZnO. The *R*_ct_ values of ZnO/GAs/ITO, ZnO/GR/ITO and ZnO/ITO were about 867 Ω, 1374 Ω, and 1678 Ω, respectively, indicating that the introduction of GAs and GR facilitates the electron transfer. However, the *R*_ct_ of ZnO/GAs/ITO was much smaller than that of ZnO/GR/ITO, indicating that GAs showed excellent conductivity than GR.

**Scheme 2:**
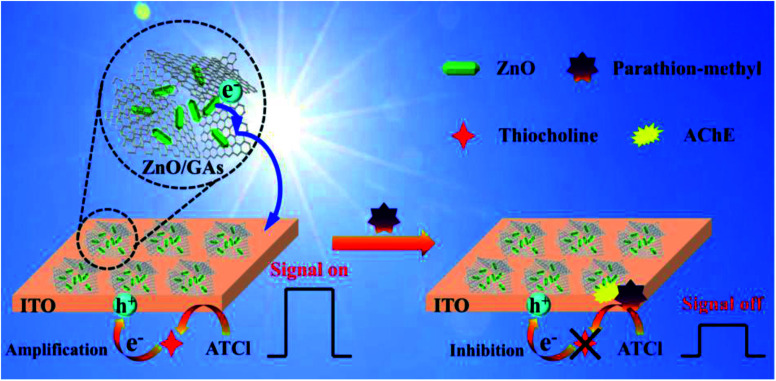
Schematic of the PEC biosensor with ZnO/GAs nanocomposites as an excellent signal-amplified platform.

**Fig. 5:**
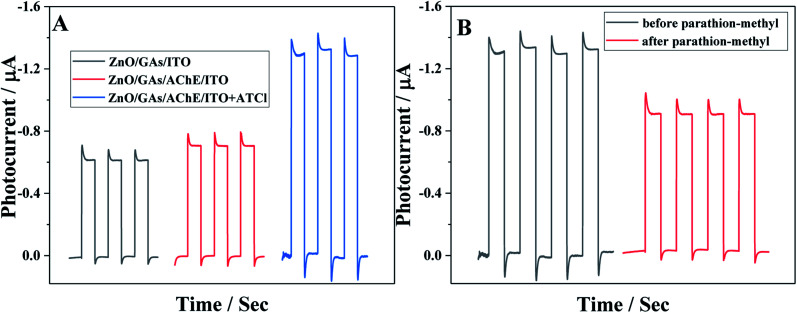
(A) The photocurrent responses of ZnO/GAs/ITO, AChE/ZnO/GAs/ITO in 0.1 mol L^−1^ PBS (pH 7.4) without and with 0.6 mmol L^−1^ ATCl; (B) the photocurrent responses of AChE/ZnO/GAs/ITO in 0.1 mol L^−1^ PBS (pH 7.4) containing 0.6 mmol L^−1^ ATCl before (a) and after (b) incubation with 0.008 μg mL^−1^ parathion-methyl.

Fig. 5A shows the photocurrent responses of the ZnO/GAs/ITO and AChE/ZnO/GAs/ITO electrodes. Compared to ZnO/GAs/ITO, the increased photocurrent of the AChE/ZnO/GAs/ITO electrode was observable with the introduction of AChE, resulting from the light-harvesting effect of AChE towards visible light and the electron or energy migration between AChE and ZnO/GAs/ITO.^40^ In the presence of ATCl, a significant enhancement in the photocurrent of the AChE/ZnO/GAs/ITO electrode was observed. The mechanism is explained is shown in Scheme 2. AChE on the surface of the ZnO/GAs/ITO electrode can hydrolyze ATCl into thiocholine and acetate. As a sacrificial electron donor, thiocholine can scavenge the holes and promote the efficiency of charge separation, resulting in a prominent increase in the photocurrent.^4,44^

To achieve ideal performance of this PEC biosensor for organophosphorus pesticide (OP) determination based on the AChE/ZnO/GAs/ITO electrode, the experimental parameters have been optimized. The optimal experimental conditions were as follows: ATCl concentration of 0.6 mmol L^−1^ (Fig. S4†), solution pH of 7.4 (Fig. S5†) and the inhibition time of 6 min (Fig. S6†), and the details were described in the ESI.†

Upon visible light irradiation, the PEC behaviors of AChE/ZnO/GAs/ITO were investigated in 0.1 M PBS (pH 7.4) containing ATCl (0.6 mmol L^−1^) before and after incubation with parathion-methyl (0.008 μg mL^−1^). As shown in Fig. 5B, the photocurrent of AChE/ZnO/GAs/ITO in 0.1 M PBS (pH 7.4) could be observed obviously. After incubation with parathion-methyl, a decreased photocurrent was observed. Based on this, the PEC biosensors in the determination of parathion-methyl can be constructed. The possible mechanism is shown in Scheme 2: when the AChE/ZnO/GAs/ITO is inhibited by parathion-methyl, the inhibitors attach to the esterase active site and inhibit the catalytic activity of AChE, leading to hindering the production of electron, resulting in a decrease in the photocurrent response.

**Fig. 6:**
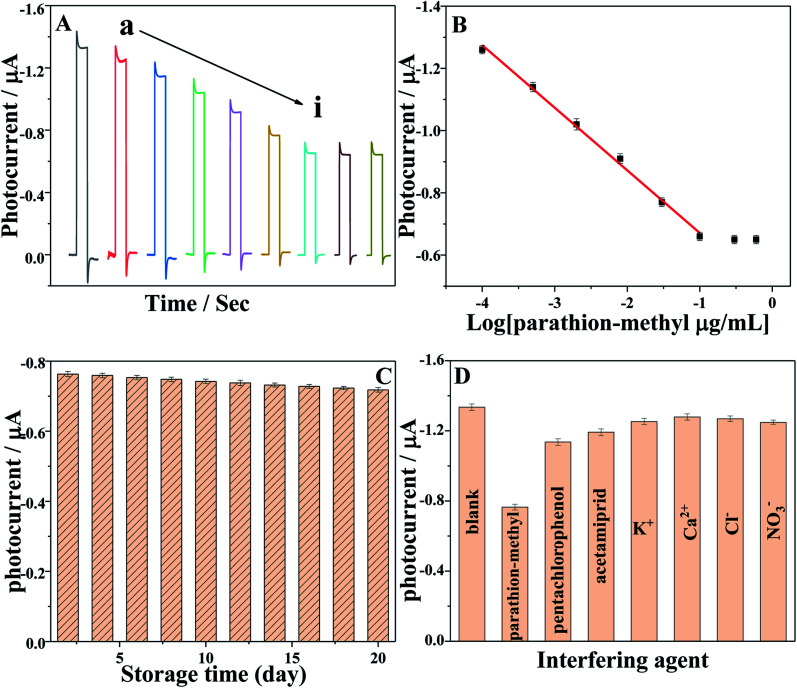
(A) The photocurrent responses of the AChE/ZnO/GAs/ITO in 0.1 mol L^−1^ PBS (pH 7.4) containing 0.6 mmol L^−1^ ATCl after incubation in (a) 0, (b) 10^−4^, (c) 5 × 10^−4^, (d) 0.002, (e) 0.008, (f) 0.03, (g) 0.1, (h) 0.3, (i) 0.6 μg mL^−1^ parathion-methyl solution; (B) calibration curve for parathion-methyl determination. Error bars: +S.D., *n* = 5; (C) stability of the PEC sensor over 20 days in 0.1 mol L^−1^ PBS (pH 7.4) containing 0.6 mmol L^−1^ ATCl after incubated with 0.03 μg mL^−1^ parathion-methyl; (D) selectivity of the PEC biosensor in 0.1 mol L^−1^ PBS (pH 7.4) containing 0.6 mmol L^−1^ ATCl in the presence of 0.03 μg mL^−1^ parathion-methyl, 0.1 μg mL^−1^ other pesticides (pentachlorophenol and acetamiprid) and 10 μg mL^−1^ inorganic ions (K^+^, Ca^2+^, Cl^−^ and NO_3_^−^).

The developed PEC biosensor was applied to monitor various of concentrations of parathion-methyl. Fig. 6A shows the PEC responses of AChE/ZnO/GAs/ITO electrodes to parathion-methyl at different concentrations (0, 10^−4^, 5 × 10^−4^, 0.002, 0.008, 0.03, 0.1, 0.3, 0.6 μg mL^−1^). The photocurrent decreased with the increase in the concentration of parathion-methyl, and the standard calibration curve for parathion-methyl detection is illustrated in Fig. 6B. The photocurrent displayed a linear range of 0.1 ng mL^−1^ to 0.1 μg mL^−1^, and the linear equation is *I* = 0.20112lg(*c* μg mL^−1^) − 0.46993 (*R*^2^ = 0.996). The detection limit was 0.03 ng mL^−1^ (S/N = 3).

### Stability and interference

The long-term stability of the PEC biosensor was investigated during 20 days (Fig. 6C), which was examined by measuring the photocurrent responses of AChE/ZnO/GAs/ITO in 0.1 M PBS (pH 7.4) containing 0.6 mmol L^−1^ ATCl after incubation with 0.03 μg mL^−1^ parathion-methyl, and the relative standard deviation (RSD) of 5.3% was obtained, indicating the reliability of the 20 days.

The selectivity of this PEC biosensor was investigated, as shown in Fig. 6D. The PEC signal for 0.6 mmol L^−1^ ATCl was compared with the signal obtained in the presence of parathion-methyl (0.03 μg mL^−1^) and the interference species, such as pentachlorophenol (1 μg mL^−1^), acetamiprid (1 μg mL^−1^), K^+^, Ca^2+^, Cl^−^ and NO_3_^−^ (0.05 M). No obvious change of the inhibition behavior can be observed, except in the presence of parathion-methyl. This result indicated that this PEC biosensor had a satisfactory selectivity.

### Real sample analysis

In order to explore the potential applicability, the proposed PEC biosensor was applied to the detection of parathion-methyl in juice and cucumber with a standard addition method. Three parathion-methyl standard solutions with known concentrations were added into the juice solution, respectively. The recoveries ranged from 98.6% to 101.6%, and satisfactory RSD values for three independent measurements were obtained. For the preparation of cucumber samples, 50 μL of different concentrations of parathion-methyl (0.1 μg mL ^−1^, 1 μg mL^−1^ and 3 μg mL^−1^) were sprayed on cucumbers peel, respectively, and it was dried for 12 h at room temperature. The parathion-methyl-exposed surface (1 × 1 cm) was swabbed with swab sticks for 1 min, which were soaked in water–methanol (1 : 1) prior to use. Then, the swabs were soaked and vortexed in 1 mL water–methanol for 2 min. The final amounts of parathion-methyl exposed to the peel surface of cucumbers were calculated to be 5 ng mL^−1^, 50 ng mL^−1^, and 150 ng mL^−1^. The recovery values of these experiments changed from 95.0% to 101.3%, as shown in Table 1. These results indicated that the as-prepared PEC biosensor had a great potential application in OP detection.

**Table 1:** Analysis of real samples with different concentrations of parathion-methyl (*n* = 3)

Samples	Added (ng mL^−1^)	Found (ng mL^−1^)	Recovery (%)	RSD (%)
Cucumber	5.00	4.87	98.7	2.7
	50.00	49.05	98.1	3.2
	150.00	150.35	100.2	4.5
Juice	5.00	5.08	101.6	4.3
	50.00	49.30	98.6	3.6
	100.00	101.3	101.3	3.2

## Conclusions

In conclusion, using a ZnO/GAs functional sensitized structure as photoelectric beacon, we designed a self-powered PEC biosensing strategy for parathion-methyl detection successfully. First, the ZnO/GAs functional sensitized structure was prepared by a facile one-pot hydrothermal method. The GAs could more efficiently improve the PEC intensity of pure ZnO than that of graphene due to its fast interfacial charge transfer. Then, the PEC signal was further enhanced by the enzymatic product. Finally, based on the inhibition effects of parathion-methyl on AChE, a PEC biosensor for parathion-methyl detection was constructed. This proposed PEC biosensor showed a satisfactory performance including a wide linear range and good selectivity. The GAs sensitized ZnO structure and efficient electron donor as a PEC signal amplification strategy opens a new avenue to improve the performances of PEC biosensors.

## Conflicts of interest

There are no conflicts to declare.

## Supplementary Material

RA-011-D1RA06339A-s001

## References

[cit1] Zhao W. W., Xu J. J., Chen H. Y. (2017). Biosens. Bioelectron..

[cit2] Ding Q., Zhu M. H., Deng H. M., Yuan R., Yuan Y. L. (2021). Biosens. Bioelectron..

[cit3] Li Y. W., Liu L., Feng J. H., Ren X., Zhang Y., Yan T., Liu X. J., Wei Q. (2020). Biosens. Bioelectron..

[cit4] Cheng W. J., Zheng Z. Y., Yang J. Y., Chen M., Yao Q. W., Chen Y. W., Gao W. H. (2019). Electrochim. Acta.

[cit5] Zhao W. W., Xu J. J., Chen H. Y. (2015). Chem. Soc. Rev..

[cit6] Li Y., Zhang N., Zhao W. W., Jiang D. C., Xu J. J., Chen H. Y. (2017). Anal. Chem..

[cit7] Guo X. X., Liu S. P., Yang M. H., Du H. T., Qu F. L. (2019). Biosens. Bioelectron..

[cit8] Zhou Q., Li G. H., Chen K. Y., Yang H., Yang M. R., Zhang Y. Y., Wan Y. K., Shen Y. F., Zhang Y. J. (2020). Anal. Chem..

[cit9] Zhao Y., Gong J., Zhang X. B., Kong R. M., Qu F. L. (2018). Sens. Actuators, B.

[cit10] Patil R. P., Mahadik M. A., Chae W. S., Choi S. H., Jang J. S. (2021). ACS Appl. Mater. Interfaces.

[cit11] Li J. A., Li X. Y., Zhao Q. D., Jiang Z., Tadé M., Wang S. B., Liu S. M. (2018). Sens. Actuators, B.

[cit12] Mao H. P., Yan Y. T., Hao N., Liu Q., Qian J., Chen S. B., Wang K. (2017). Sens. Actuators, B.

[cit13] Wang X. W., Zhou C. X., Yin L. C., Zhang R. B., Liu G. (2019). ACS Sustainable Chem. Eng..

[cit14] Zhao L. F., Ji J. J., Shen Y. F., Wu K. Q., Zhao T. T., Yang H., Lv Y. Q., Liu S. Q., Zhang Y. J. (2019). Chem.–Eur. J..

[cit15] Yang H., Zhou Q., Fang Z. Z., Li W., Zheng Y. J., Ma J., Wang Z., Zhao L. F., Liu S. Q., Shen Y. F., Zhang Y. J. (2021). Chem.

[cit16] Liu H., Sun Q., Xing J., Zheng Z. Y., Zhang Z. Y., Lü Z. Q., Zhao K. (2015). ACS Appl. Mater. Interfaces.

[cit17] Kang Z., Gu Y. S., Yan X. Q., Bai Z. M., Liu Y. Y., Liu S., Zhang X. H., Zhang Z., Zhang X. J., Zhang Y. (2015). Biosens. Bioelectron..

[cit18] Rani M., Tripathi S. K. (2016). Renewable Sustainable Energy Rev..

[cit19] Buonsanti R., Llordes A., Aloni S., Helms B. A., Milliron D. J. (2011). Nano Lett..

[cit20] Pang X. H., Zhang Y., Pan J. H., Zhao Y. X., Chen Y., Ren X., Ma H. M., Wei Q., Du B. (2016). Biosens. Bioelectron..

[cit21] Zhu L. Y., Li H., Xia P. F., Liu Z. R., Xiong D. H. (2018). ACS Appl. Mater. Interfaces.

[cit22] Ho W. K., Chen J. S., Wu J. J. (2021). ACS Sustainable Chem. Eng..

[cit23] Wang Y. J., Wang F. M., He J. (2013). Nanoscale.

[cit24] Yan Y. T., Li H. N., Liu Q., Hao N., Mao H. P., Wang K. (2017). Sens. Actuators, B.

[cit25] Xu Y. X., Lin Z. Y., Huang X. Q., Liu Y., Huang Y., Duan X. F. (2013). ACS Nano.

[cit26] Xu Y. X., Sheng K. X., Li C., Shi G. Q. (2010). ACS Nano.

[cit27] Yang F., Wang G., Mei T., Li J. H., Wang J. Y., Wang X. X. (2017). ACS Sustainable Chem. Eng..

[cit28] Ghosh D., Lim J., Narayan R., Sang O. K. (2016). ACS Appl. Mater. Interfaces.

[cit29] Qiao H., Huang Z. Y., Liu S. Q., Tao Y., Zhou H., Li M. Y., Qi X. (2019). J. Phys. Chem. C.

[cit30] Zhang M. M., Wang Y., Pan D. H., Li Y., Yan Z. X., Xie J. M. (2017). ACS Sustainable Chem. Eng..

[cit31] Sha C. H., Cheng J. P., Mao H. Y., Pan X. H., Ye Z. Z., Lu B. (2018). Electrochim. Acta.

[cit32] Fan L. S., Zhang Y., Zhang Q., Wu X., Cheng J. H., Zhang N. Q., Feng Y. J., Sun K. N. (2016). Small.

[cit33] Cai R., Wu J. G., Sun L., Liu Y. J., Fang T., Zhu S., Li S. Y., Wang Y., Guo L. F., Zhao C. E., Wei A. (2016). Mater. Des..

[cit34] Yue H. Y., Zhang H., Huang S., Lin X. Y., Gao X., Chang J., Yao L. H., Guo E. J. (2017). Biosens. Bioelectron..

[cit35] Hou T., Zhang L. F., Sun X. Z., Li F. (2016). Biosens. Bioelectron..

[cit36] Hummers W. S., Offeman R. E. (1958). J. Am. Chem. Soc..

[cit37] Shen J. F., Hu Y. Z., Shi M., Lu X., Qin C., Li C., Ye M. X. (2009). Chem. Mater..

[cit38] Bai S. L., Hu J. W., Li D. Q., Luo R. X., Chen A. F., Liu C. C. (2011). J. Mater. Chem..

[cit39] Liu X., Sun J. B., Zhang X. T. (2015). Sens. Actuators, B.

[cit40] Yang S., Guo X., Chen P., Xu D. W., Qiu H. F., Zhu X. Y. (2019). J. Alloys Compd..

[cit41] Wang C. X., Shi P. H., Cai X. D., Xu Q. J., Zhou X. J., Zhou X. L., Yang D., Fan J. C., Min Y. L., Ge H. H., Yao W. F. (2016). J. Phys. Chem. C.

[cit42] Kokulnathan T., Ahmed F., Chen S. M., Chen T. W., Hasan P. M. Z., Bilgrami A. L., Darwesh R. (2021). ACS Appl. Mater. Interfaces.

[cit43] Zhang Z. Y., Xiao F., Guo Y. L., Wang S., Liu Y. Q. (2013). ACS Appl. Mater. Interfaces.

[cit44] Song J., Wu S., Xing P. P., Zhao Y. Q., Yuan J. L. (2018). Anal. Chim. Acta.

