# Supplementary material for: A one-step hydrothermal route to fabricate a ZnO nanorod/3D graphene aerogel-sensitized structure with enhanced photoelectrochemistry performance and self-powered photoelectrochemical biosensing of parathion-methyl
Source: RSC Adv. 2021 Nov 3;11(56):35644–52. doi: 10.1039/d1ra06339a (PMC9043228; doi:10.1039/d1ra06339a)
Supplement: RA-011-D1RA06339A-s001 [file RA-011-D1RA06339A-s001.pdf]

Electronic Supplementary Material (ESI) for RSC Advances.
This journal is © The Royal Society of Chemistry 2021

Supporting Information

One-step hydrothermal route to fabricate ZnO nanorods/3D graphene aerogel sensitized structure with enhanced Photoelectrochemistry performance and self-powered Photoelectrochemical biosensing of parathion-methyl

Yuting Yan[1*], Qian Li[2],Qirui Wang[3], Hanping Mao[1*]

[1]*School of Agricultural Engineering, Jiangsu University, Zhenjiang, 212013, People ' s Republic of China*

[2] *School of food Science and Engineering,Yangzhou University, Yangzhou, 225127, People ' s Republic of China*[3] *School of Electrical and information Engineering, Changzhou Institute of Technology, Changzhou,213022, People ' s Republic of China*

---

*Corresponding author. Tel.: +86 511 88797338; fax: +86 51188797338.

E-mail address: yanyuting@ujs.edu.cn; maohp@ujs.edu.cn

Fig.S1. SEM image of GAs.

Fig.S2. EDS (A), EDX elemental mapping of Zn (B), C (C) and O (D) of the ZnO/GAs nanocomposites.

Fig.S3.XRD pattern of GO.

Fig.S4. Effects of ATCl concentration on the photocurrent response of the AChE/ZnO/GAs/ITO.

Fig.S5. Effect of pH values on the photocurrent response of AChE/ZnO/GAs/ITO.

Fig.S6. Effects of inhibition time of 0.008μg mL^{-1} parathion-methyl on the photocurrent response of the AChE/ZnO/GAs/ITO

Fig.S1

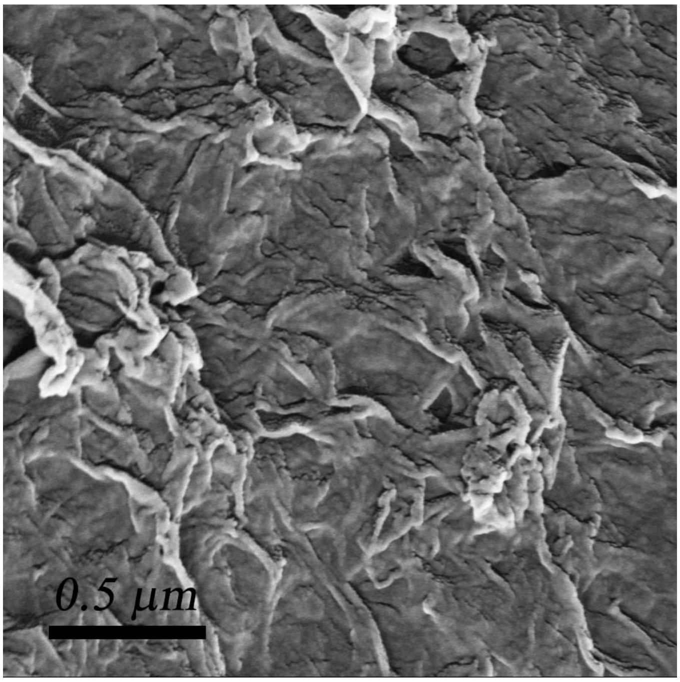

Fig.S2

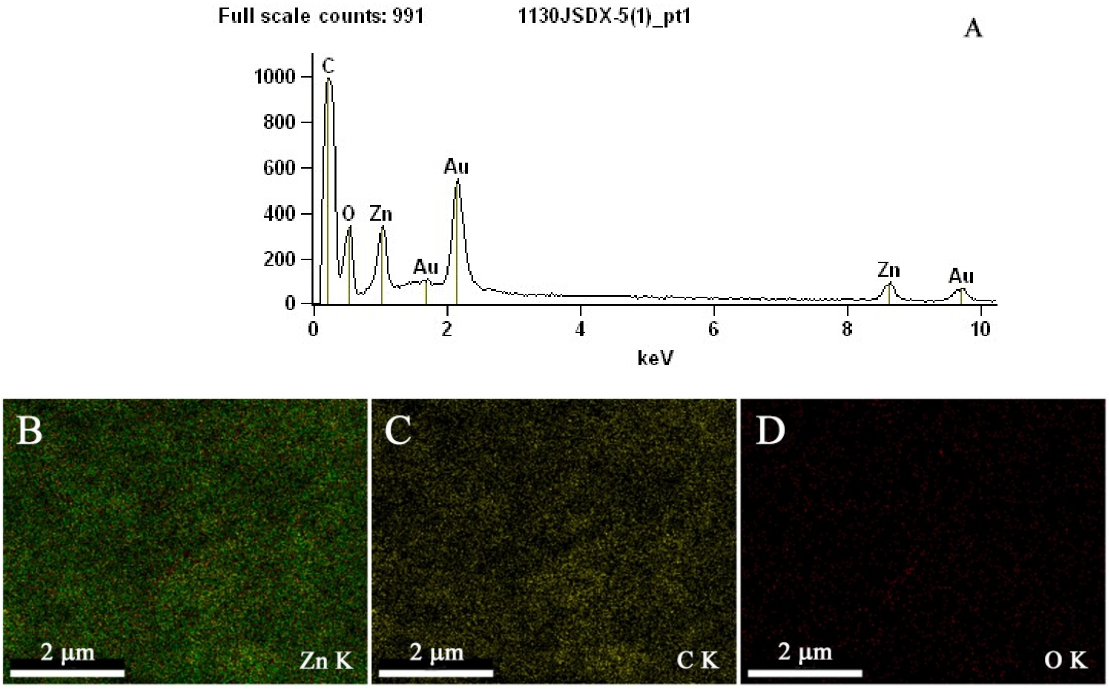

Full scale counts: 991
1130JSDX-5(1)_pt1
A
1000
800
600
400
200
0
C
O
Zn
Au
Au
Zn
Au
0
2
4
6
8
10
keV
B
2 μm
Zn K
C
2 μm
C K
D
2 μm
O K

Fig.S3

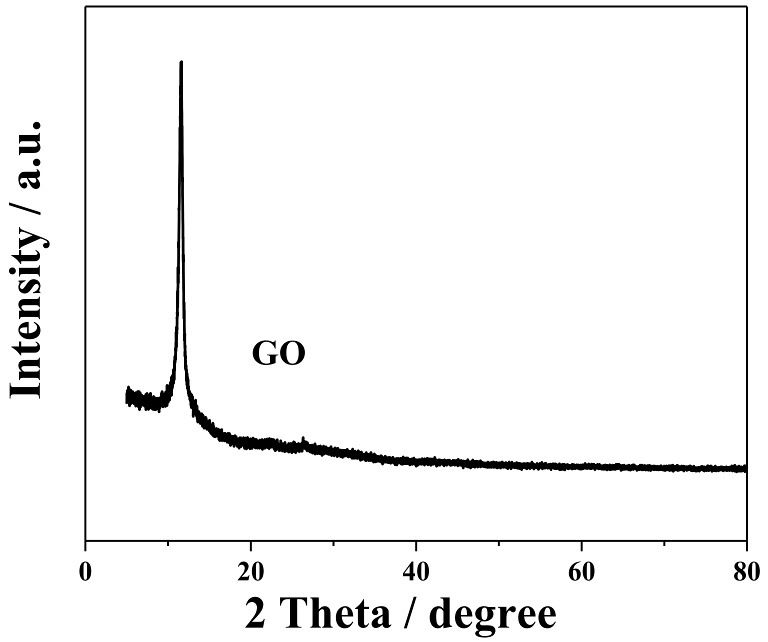

Fig.S4

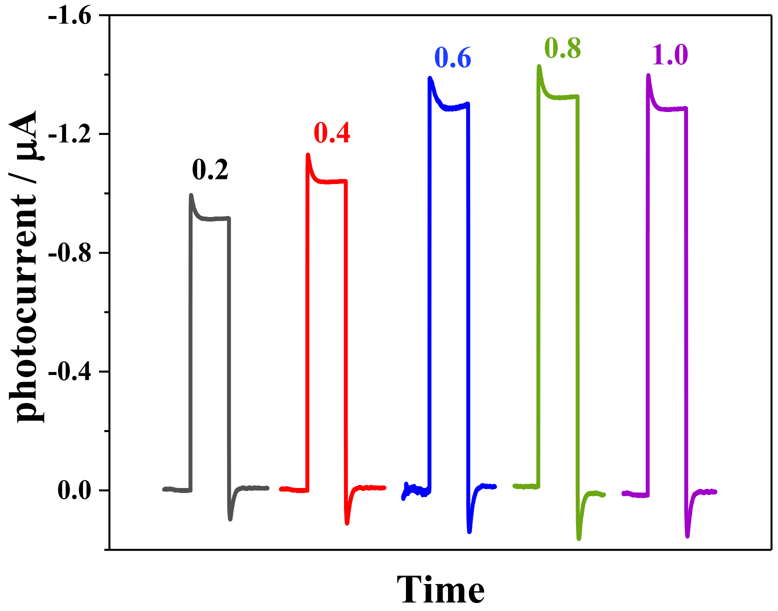

Fig.S5

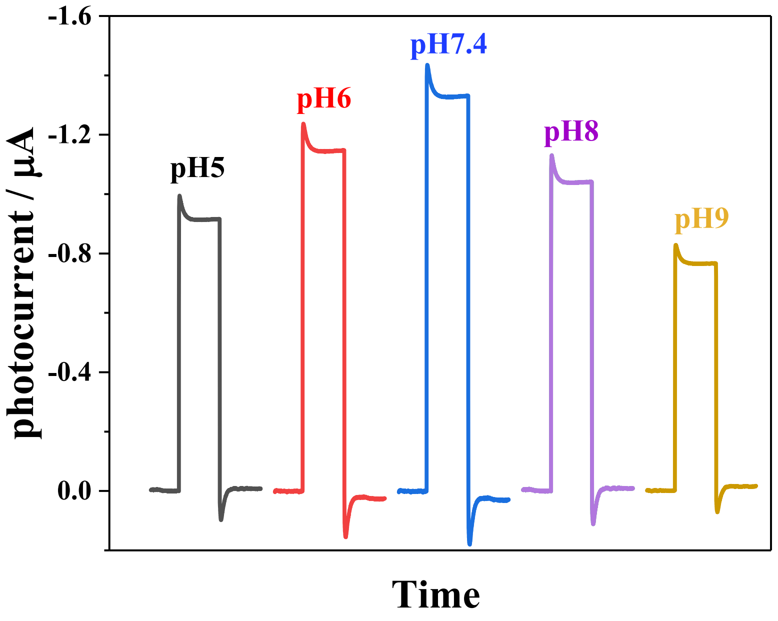

Fig.S6

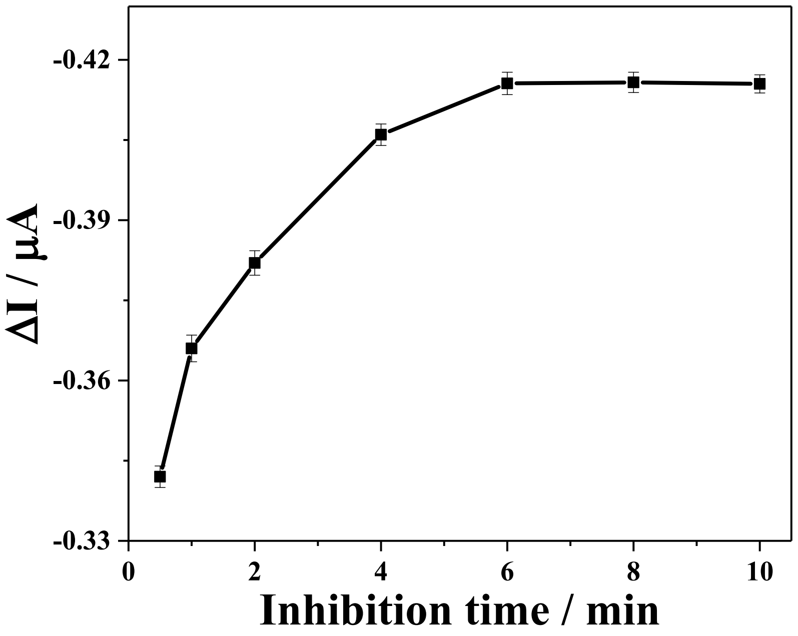

The concentration of ATCl was one of the most influential parameters. When the concentration of ATCl is too low, the photocurrent generated by the enzyme electrode is too small that the determination of OPs is hardly feasible. However, too high concentration would cause all the active centers of AChE being occupied by the substrate and insensible to the inhibitor. Thus, the effect of ATCl concentration on the photocurrent responses of the AChE/ZnO/GAs/ITO film was investigated in our work (Fig.S4). The photocurrent increased gradually with the increase of the ATCl concentration, and when the concentration of ATCl reached 0.6 mM, the photocurrent increased slowly and tended to a stable signal. Thus, the concentration of 0.6mMATCl is an optimized value in this experiment.

The effect of pH on the PEC behavior of AChE/ZnO/GAs/ITO electrode was performed in 0.1 M PBS with the pH rang of 5.0 ~ 9.0 in the presence of 0.6 mM ATCl. As shown in Fig. S5, the PEC intensity increased with the increasing of pH from 5.0 to 7.4, and then dropped at high pH from 7.4 to 9.0, indicating that the AChE/ZnO/GAs/ITO electrode exhibited a relatively stable PEC signal in a wide pH range. Thus, pH 7.4 was chosen as the optimal pH value in the following experiments to keep the bioactivity of AChE on the AChE/ZnO/GAs/ITO electrode.

Inhibition time is an important influential parameter in pesticide analysis. So the dependence of inhibition time on the parathion-methyl inhibition was also investigated, as shown in Fig.S6. Parathion-methyl displayed an increasing inhibition on AChE with the immersion time increased. When the immersion time was longer than 6 min, the inhibition tended to a stable value, which indicated that the binding interaction with active target groups in the enzyme reached saturation. Therefore, 6 min inhibition time was used in our

experiment.
